# Impact of Pancreatic Rat Islet Density on Cell Survival during Hypoxia

**DOI:** 10.1155/2016/3615286

**Published:** 2015-12-28

**Authors:** A. Rodriguez-Brotons, W. Bietiger, C. Peronet, J. Magisson, C. Sookhareea, A. Langlois, C. Mura, N. Jeandidier, M. Pinget, S. Sigrist, E. Maillard

**Affiliations:** ^1^UMR DIATHEC, EA 7294, Centre Européen d'Etude du Diabète, Université de Strasbourg, Fédération de Médecine Translationnelle de Strasbourg, Bld René Leriche, 67200 Strasbourg, France; ^2^Defymed, avenue Dante, 67200 Strasbourg, France; ^3^Structure d'Endocrinologie, Diabète-Nutrition et Addictologie, Pôle NUDE, Hôpitaux Universitaires de Strasbourg (HUS), 67000 Strasbourg, France

## Abstract

In bioartificial pancreases (BP), the number of islets needed to restore normoglycaemia in the diabetic patient is critical. However, the confinement of a high quantity of islets in a limited space may impact islet survival, particularly in regard to the low oxygen partial pressure (PO_2_) in such environments. The aim of the present study was to evaluate the impact of islet number in a confined space under hypoxia on cell survival. Rat islets were seeded at three different concentrations (150, 300, and 600 Islet Equivalents (IEQ)/cm^2^) and cultured in normal atmospheric pressure (160 mmHg) as well as hypoxic conditions (15 mmHg) for 24 hours. Cell viability, function, hypoxia-induced changes in gene expression, and cytokine secretion were then assessed. Notably, hypoxia appeared to induce a decrease in viability and increasing islet density exacerbated the observed increase in cellular apoptosis as well as the loss of function. These changes were also associated with an increase in inflammatory gene transcription. Taken together, these data indicate that when a high number of islets are confined to a small space under hypoxia, cell viability and function are significantly impacted. Thus, in order to improve islet survival in this environment during transplantation, oxygenation is of critical importance.

## 1. Introduction

Islet transplantation is a minimally invasive therapy recommended for patients with brittle diabetes. Over the last fifteen years, the outcome of this therapy has improved, with a 3-year graft survival rate of 44% [1] as well as a decreased insulin requirement even after partial loss of the graft [2–6]. However, the main advantage of this procedure for diabetic patients is that even if they must be put back on insulin, there is a residual protective effect from hypoglycemic unawareness episodes thanks to a basal level of C-peptide (<0.3 g/L) [1]. Although this treatment option appears to have a number of positive consequences, the negative effects and limitations of the transplant, including the pancreatic requirements, immune suppressive regimen, low rate of insulin independence, short lifespan of the graft, and the recurrence of autoimmunity, have restricted the use of this therapy, particularly when treating children [6, 7].

To address these issues, bioartificial pancreases that encapsulate the cells in a selective shield which lets nutrients and insulin cross the barrier while stopping immune cells and effectively hiding the graft from the host are currently being developed [2, 6]. To immune-isolate the islet cells in this manner, one of two strategies is typically utilized, microencapsulation or macroencapsulation. For the first, one islet to four islets are encapsulated in a microsphere of polymer [2]. This polymer facilitates the exchange of oxygen and nutrients but makes it difficult to retrieve the graft posttransplantation. In contrast, for macroencapsulation, islets are gathered in one or a few distinct devices (e.g., a sheet of polymer, cylindrical device, perfusion device, macrobeads, or selective membrane [2–6, 8]), which allow the grafted cells to be easily removed, providing an additional advantage in the favor of macroencapsulation from a regulatory point of view for clinical application [9, 10]. However, the gathering of these cells can be detrimental as a lower level of oxygen is available in the device and the islets are confined to a much more limited area, potentially increasing the local consumption of oxygen and amplifying the hypoxic conditions surrounding the cells [6].

Physiologically, islets in a normal pancreas make up less than 2% of the whole organ but use approximately 10% of the total oxygen supply. These cells are highly vascularized and not physiologically prepared to face hypoxia. During islet transplantation in the liver, an oxygen partial pressure (PO_2_) of 5–10 mmHg has been shown to be enough for their survival [11], but this likely only reflects the level necessary when the islets are directly connected to the vascular system. In a bioartificial pancreas, islets cannot be vascularized as this will disrupt the immune-isolation characteristics necessary for their function. Thus, in these devices, oxygen can only be accessed via membrane diffusion, and PO_2_ in a macroencapsulation device is around 15 mmHg (subcutaneously or intraperitoneally [12–15]). It is unlikely that this level is high enough for the cells to function efficiently.

The consequences of hypoxia on islets, including impaired survival and function [16, 17] and increased inflammation [18] associated with the recruitment and activation of host macrophages and leukocytes in the implantation site [19], are well established. Inflammatory molecules such as cytokines and chemokines promote insulitis and *β*-cell destruction together with an increased production of reactive oxygen species (ROS). The main strategy used to counteract hypoxia-induced loss of islets is to overload the devices with cells with the hope that a few will survive and help reverse the effects of diabetes. In fact, experiments performed with the TheraCyte device, which uses 1,000 islets per device, have demonstrated a 6-month survival period for the graft in an immunized rat [20]. Unfortunately, this previous study only focused on islet function 1 month after transplantation. Furthermore, no data are available concerning islet oxygenation, and the authors have only assumed that the vascularization surrounding the device was able to bring enough oxygen for islet survival. Notably, one of the primary concerns, with increasing the number of islets in the confined environment of the device, is that even more oxygen will be consumed [21, 22], further exacerbating the localized hypoxia.

The number of islets transplanted is also depending on the size of the patient. For instance, a patient that weighs 50 kg would require a graft of 500,000 Islet Equivalents (IEQ). In order to maintain a sufficient number of functioning islet cells, the typical preconized culture density is 150 Islet Equivalents (IEQ)/cm^2^ [21], which means that to transplant this small patient, a single 3,333 cm^2^ device or 500 small 6.67 cm^2^ devices would need to be inserted, which is impossible. Thus, it is essential to increase the number of survival cells/cm^2^.

In the present study, we investigated the effects of islet density in a cellular context that mimics that found in smaller sized bioartificial pancreases using under 15 mmHg of oxygen. In doing so, we sought to establish the effects of hypoxic islet confinement on islet survival, function, and inflammatory potency in order to develop new strategies to improve the outcome of islet transplantation in patients with diabetes.

## 2. Materials and Methods

### 2.1. Islet Cell Isolation and Culture

#### 2.1.1. Animals

Male Wistar rats were supplied by Janvier Laboratory (Le Genes St Isle, France). All rats were housed in pathogen-free conditions, in standard collective cages, in a temperature-controlled room (23 ± 1°C) with a 12-hour light/12-hour darkness cycle. They were fed SAFE-A04 (Villemoisson-sur-Orge, France) food and water* ad libitum*. All experiments were performed according to the National Institutes of Health and local ethical committee (CREMEAS) guidelines (authorization number: C67-482-28).

#### 2.1.2. Islet Isolation

Pancreatic islets were isolated from adult Wistar rat pancreas (weight: 200–250 g) using standard collagenase (Sigma-Aldrich, St. Louis, MO, USA) digestion and Ficoll (Eurobio, Les Ulis, France) purification. Islets were cultured in Medium 199 containing 5.5 mM glucose (Gibco, Life Technologies, Paisley, Scotland) supplemented with 10% heat-inactivated fetal bovine serum (FBS, Sigma-Aldrich), and 1% antibiotic/antimicotic solution (ABAM; 100 units/mL of penicillin, 100 *μ*g/mL of streptomycin, and 0.25 *μ*g/mL of Fungizone; Gibco). After culturing for 24 hours in 25 cm^2^ flasks (Cellstar, Greiner Bio-One GmbH, Frickenhausen, Germany), three different islet densities (150, 300, and 600 IEQ/cm^2^) were seeded in 48-well plates (Greiner). The 150 IEQ/cm^2^ density was considered as the control condition in these experiments. Islets were cultured at the specified density for 24 hours in a humidified incubator (StemCell Technologies, Canada) at 37°C under normal atmosphere (PO_2_: 160 mmHg) as well as hypoxia conditions (PO_2_: 15 mmHg).

### 2.2. Oxygen Measurements

Oxygen measurements were carried out with a four-channel fiber-optic oxygen meter (Oxy-4) and noninvasive oxygen sensors (Presens, Germany). Briefly, oxygen acts as a dynamic fluorescence quencher of a luminophore in a polymer matrix. Spots, made of this polymer matrix, were glued onto the bottom of each culture well (Presens, Germany), allowing noninvasive measurements of oxygen to be taken through the plastic from the outside. An optic fibre connected to the OXY 4 oxygen meter was guided and positioned from the outside of the chamber to the spot. The data are expressed in mmHg and were taken at time 0 (t0) before placing the plates into the incubators as well as after the 24 h incubation period.

### 2.3. Islet Viability

Viability of 10 islets from each condition was analyzed by fluorescein diacetate/propidium iodide staining (FDA/PI, Sigma) by three independent investigators. The ratio of green to red cells provided the percentage of islet viability. Images were obtained on a Nikon Eclipse 50i microscope with Nis-Element-BR software (Nikon, Amstelveen, Netherlands).

### 2.4. ATP Levels

A total of 50 islets from each condition were handpicked and ATP was extracted with mammalian cell lysis solution. Levels of ATP were assessed using an ATPlite kit (PerkinElmer Inc., Waltham, MA, USA) following the manufacturer's instructions. Results are expressed as *μ*M/50 islets.

### 2.5. Islet Functionality

A subset of islets (*n* = 10) from each experimental condition were washed extensively and incubated in Krebs Ringer bicarbonate (KRB) solution with 10% FBS and 4.4 mmol/L of glucose (Sigma). Islets were then stimulated with KRB solution containing 10% FBS and 22.6 mmol/L of glucose. Each incubation step was performed for 90 min at 37°C in a humidified 5% CO_2_ atmosphere. Supernatants were then collected and stored at −80°C. Insulin measurements were performed using a rat insulin enzyme linked immunosorbent assay (ELISA) kit (Mercodia, Uppsala, Sweden). Results are expressed as a stimulated index (SI) defined as the ratio of stimulated versus basal insulin secretion.

### 2.6. Real-Time PCR

Total cellular RNA was extracted from approximately 400 islets from each condition using a Qiagen RNeasy Mini Kit (Qiagen, CA, USA), following the manufacturer's instructions. Extracted RNA was reverse-transcribed with a RT^2^ First Strand Kit (Qiagen), according to the manufacturer's instructions. The resulting cDNAs were diluted 1/100 in RNase-free water. Real-time PCR reactions were performed on a MyiQ Real-Time PCR System (Qiagen) using a QuantiTect SYBR Green PCR kit (Qiagen). Mouse 60S acidic ribosomal protein P1 (Rplp1), mouse peptidylprolyl isomerase A (Ppia), and mouse RNA ribosomal 1 (Rnr1) were chosen as the housekeeping genes (Table 1), and data were analyzed using the ΔΔCt method. The primers for hypoxia-inducible factor 1 alpha (Hif-1*α*), prostaglandin-endoperoxide synthase 2 (Ptgs2/Cox-2), and heme oxygenase 1 (Hmox1/Ho-1) used in this study were all purchased from Qiagen and are listed in Table 1. Amplification was run using a maximum of 35 cycles.

### 2.7. Protein Extraction

The total protein content was extracted from 300 islets from each condition using M-PER Mammalian Protein Extraction Reagents and HALT Protease and phosphatase inhibitor cocktail (Thermo Fisher Scientific, Illkirch-Graffenstaden, France). Protein concentration was determined using the Bradford micromethod (Bio-Rad, Life Science Group, Marnes-la-Coquette, France) and is expressed as mg/mL.

Caspase 3 activation in these cytosolic protein extracts of the islets was assessed with a Quantikine ELISA kit (R&D Systems, Minneapolis, MN, USA). Results are expressed in ng/mg of protein.

### 2.8. HIF-1*α* Activation (ELISA)

Hif-1*α* activation was quantified by TransAM HIF-1 (Active Motif, Belgium) following the manufacturer's instructions. 20 *μ*g of the total protein extraction was used to assess HIF-1*α*. HIF dimers bind specifically to hypoxia response element (HRE) immobilized in the 96-well plate. HIF dimers are detected by HIF-1*α* antibody (dilution: 1/500) and a secondary antibody conjugated to HRP (dilution: 1/1000). The results are expressed in OD.

### 2.9. Inflammation

An aliquot of the culture medium was used to measure the concentration of secreted interleukin- (IL-) 6 (R&D Systems, Minneapolis, MN, USA) under each condition using an ELISA kit. Results are expressed in pg/mL/IEQ.

### 2.10. Histology

A total of 300 islets from each condition were harvested, washed, and snap-frozen in optimal cutting temperature compound (Tissue O.C.T. Labonord, Templemars, France) and then sliced in 12 *μ*m sections. Apoptotic cells were stained using a Terminal deoxynucleotidyl transferase dUTP Nick End Labeling (TUNEL) assay (Millipore, Molsheim, France) and observed by florescence microscopy.

Hypoxic staining was performed using a Hypoxyprobe-1 Kit (HPI Inc. Burlington, MA, USA). Solid pimonidazole HCl was added in the islet cell cultures (150 *μ*M). After 24 h culture, islets were fixed with 2% paraformaldehyde (PFA) for 10 min and then incubated with primary and secondary antibodies. The antibodies used in this study include anti-mouse immunoglobulin (IgG_1_, 1 : 50 dilution; HPI, Inc.) and Alexa Fluor 488 goat anti-mouse IgG (dilution 1 : 200, Life technologies, Carlsbad, CA, USA).

### 2.11. Statistical Analysis

Statistical analyses were performed using Statistics software (StatSoft, Maisons-Alfort, France). Results were analyzed with one-way analysis of variance (ANOVA) followed by post hoc Tukey honest significant difference (HSD) testing for the parametric data or Kruskal-Wallis for the nonparametric analysis. Results are expressed as mean ± SEM. A *p* value less than 0.05 was considered statistically significant.

## 3. Results

### 3.1. Oxygen Measurement

At t0, the PO_2_ was found to be relatively simiar for all three culture densities (150, 300, and 600 IEQ/cm^2^) under both normal and hypoxic conditions (Table 2). However, after 24 hours of exposure to hypoxia (PO_2_: 15 mmHg), the PO_2_ of the control islet cultures (150 IEQ/cm^2^) dropped to 14.3 ± 6.2 mmHg compared to 121 ± 9.7 mmHg under normal conditions. We also observed a decrease in the PO_2_ in the cells cultured at the 300 and 600 IEQ/cm^2^ densities (99.5 ± 12.7 mmHg and 80.5 ± 8.7 mmHg, resp.) compared to the control after being in culture for 24 hours under normoxic conditions. Furthermore, the hypoxia-induced drop in the PO_2_ observed in the control islets was exacerbated when the cell density was increased to 300 and 600 IEQ/cm^2^ (10.5 ± 5.1 mmHg and 7.6 ± 3.2 mmHg, resp.).

### 3.2. Islet Viability

**Figure 1:**
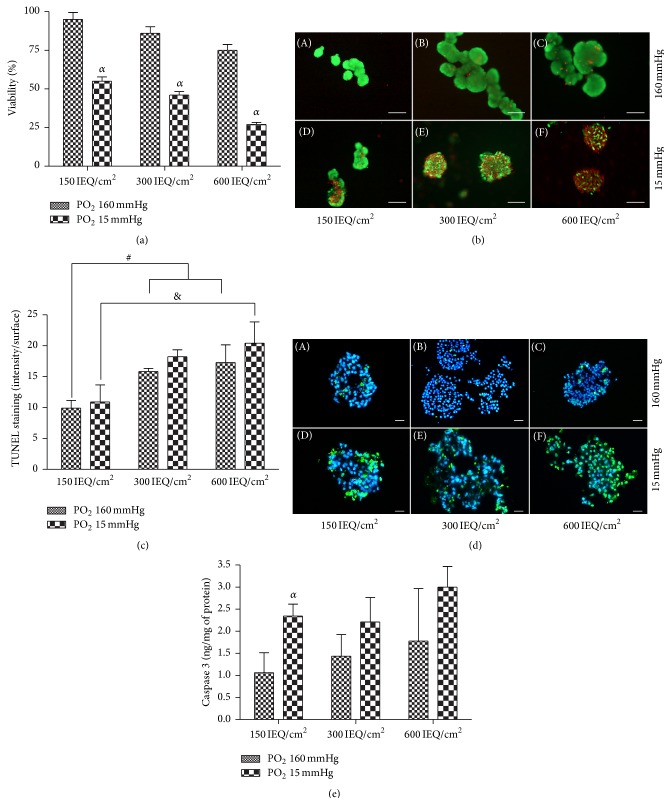
Islet viability. (a) Islet viability at each cell density after 24 hours in culture under both normoxic (PO_2_ 160 mmHg) and hypoxic (PO_2_ 15 mmHg) conditions. (b) Representative fluorescein diacetate/propidium iodide (FDA/PI) stained images of islets cultured at 150, 300, or 600 IEQ/cm^2^ under normoxia and hypoxia. Green staining (FDA) indicates that the cells are alive, while red staining (PI) indicates that the cells are dead. Scale bars = 100 *μ*m. (c) Islet apoptosis at each cell density after 24 hours in culture under both normoxic (PO_2_ 160 mmHg) and hypoxic (PO_2_ 15 mmHg) conditions. (d) Representative images of TUNEL stained cells cultured at 150, 300, or 600 IEQ/cm^2^ under normoxia and hypoxia. Blue staining highlights the cell nuclei (DAPI), and the green staining indicates apoptosis. Scale bars = 50 *μ*m. (e) Caspase-3 activation at each cell density after 24 hours in culture under both normoxic (PO_2_ 160 mmHg) and hypoxic (PO_2_ 15 mmHg) conditions. ^*α*^ *p* < 0.05 compared to the control, ^*ααα*^ *p* < 0.001 compared to 600 IEQ/cm^2^ normoxia.

Under hypoxic conditions, the viability of the islets cultured for 24 hours at a density of 150 IEQ/cm^2^ was significantly decreased compared to those under normal atmosphere (Figure 1(a)). A similar trend was observed at the higher densities, with the greatest difference being observed at 600 IEQ/cm^2^. Representative FDA/PI stained images of the normal and hypoxic cells at each density are shown in Figure 1(b). These data are supported by a significant increase in Caspase 3 protein expression (Figure 1(c); *p* < 0.05) as well as an observed increase in TUNEL staining (Figure 1(d)) in the hypoxia treated cells compared to the normoxia treated cells. Taken together, these data indicate that islet density/confinement did not have a significant effect on the percentage of viable cells when cultured in normoxic conditions but does appear to intensify the effects of hypoxia.

### 3.3. Hypoxia Marker Expression

**Figure 2:**
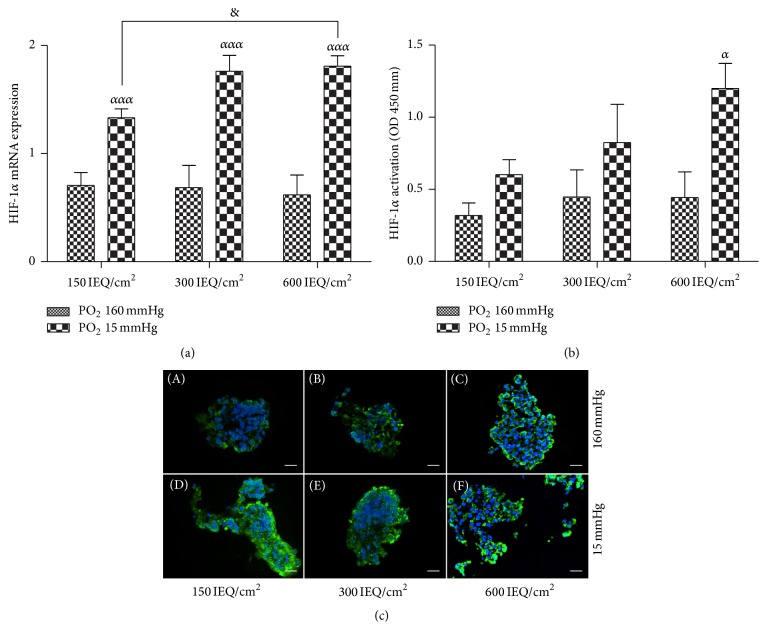
Hypoxia marker expression. (a) Levels of hypoxia-inducible factor 1 alpha (HIF-1*α*) mRNA expression after 24 hours in culture under normoxia (PO_2_ 160 mmHg) and hypoxia (PO_2_ 15 mmHg) at 150, 300, and 600 IEQ/cm^2^. (b) Translocation and activation of HIF-1*α* protein were performed after 24 hours in each density (150, 300, and 600 IEQ/cm^2^) under normoxia and hypoxia conditions. (c) Representative images of hypoxyprobe stained cells at 150, 300, and 600 IEQ/cm^2^ under normoxia and hypoxia. Blue staining highlights the cell nuclei (DAPI), and the green staining indicates pimonidazole (hypoxia marker) expression. Scale bars = 50 *μ*m. ^*ααα*^ *p* < 0.001 compared to the same concentration under normoxic conditions, ^&^ *p* < 0.05 compared to 150 IEQ/cm^2^ under hypoxic conditions, and ^*α*^ *p* < 0.05 compared to the control.

Not surprisingly, HIF-1*α* mRNA expression (Figure 2(a)) and hypoxyprobe staining (Figure 2(c)) were significantly increased in the control islets cultured under hypoxic conditions compared to those cultured under normoxic conditions. Furthermore, the density/confinement of the islets also appeared to have a significant impact on the HIF-1*α* mRNA expression and hypoxyprobe staining when the cells were cultured under hypoxic conditions. A correlation between the increase in density and the activation of HIF-1*α* protein (Figure 2(b)) appeared under hypoxia and became significant for 600 IEQ/cm^2^ (*p* < 0.05) compared to normoxic conditions. In fact, the levels of these hypoxia markers appeared to increase with the increasing level of hypoxia, indicating a particularly significant increase in the cells cultured at 600 IEQ/cm^2^ (*p* < 0.05) compared to the cell cultured at 150 IEQ/cm^2^ under hypoxic conditions.

### 3.4. Islet Functionality

**Figure 3:**
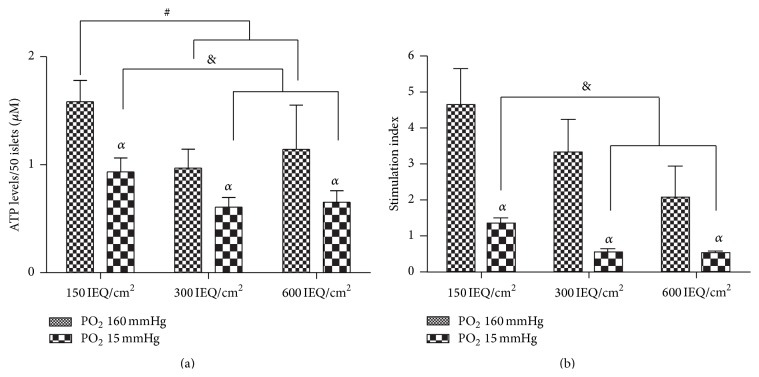
Islet functionality. (a) ATP levels were measured after 24 hours of culture at the 150, 300, and 600 IEQ/cm^2^ under normoxic (PO_2_ 160 mmHg) and hypoxic (PO_2_ 15 mmHg) conditions. (b) Insulin response to glucose stimulation was measured after 24 hours in culture using a stimulation index. Each density (150, 300, and 600 IEQ/cm^2^) was evaluated under both normoxic (PO_2_ 160 mmHg) and hypoxic (PO_2_ 15 mmHg) conditions. ^*α*^ *p* < 0.05 compared to the control, ^#^ *p* < 0.05 compared to 150 IEQ/cm^2^ under normoxia, and ^&^ *p* < 0.05 compared to 150 IEQ/cm^2^ under hypoxia.

Islet function, in terms of ATP levels in the cell, was also evaluated. These data demonstrate that, compared to normoxia, hypoxia induced a significant decrease in the ATP content regardless of cell culture density (*p* < 0.05). However, this decrease in ATP content was significantly more prominent for the cells culture in the more confined densities compared to those cultured at 150 IEQ/cm^2^ (*p* < 0.05) (Figure 3(a)). These results were also supported during our analysis of insulin secretion. We observed a significant loss of function when the cells were cultured under hypoxia compared to normoxia (*p* < 0.05). At 300 IEQ/cm^2^ and 600 IEQ/cm^2^, under normoxic conditions we observed a slight decrease in the stimulation index for both densities compared to the control density; however, these islets were still considered functional as their stimulation indices were around or over 2. Under hypoxia, confinement tended to emphasize the loss of function observed in the control density, with the indices being significantly reduced to less than 1 for both the 300 IEQ/cm^2^ and 600 IEQ/cm^2^ densities compared to 150 IEQ/cm^2^ (Figure 3(b)).

### 3.5. Inflammation

**Figure 4:**
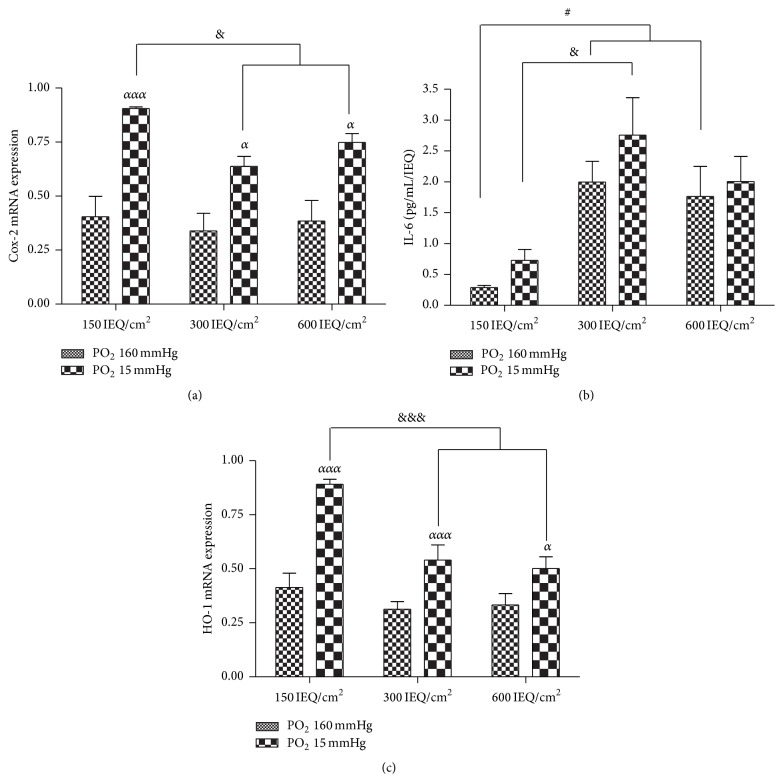
Inflammation. (a) Levels of Cox-2 mRNA expression in islets after 24 hours in culture at 150, 300, and 600 IEQ/cm^2^ under normoxic (PO_2_ 160 mmHg) and hypoxic (PO_2_ 15 mmHg) conditions. (b) Levels of Ho-1 mRNA expression after 24 hours in culture under the specified conditions. (c) IL-6 secretion was measured in islet medium after 24 hours in culture under the specified conditions. ^*α*^ *p* < 0.05 compared to the control, ^*ααα*^ *p* < 0.001 compared to 150 IEQ/cm^2^ under normoxic conditions, ^#^ *p* < 0.05 compared to 150 IEQ/cm^2^ under normoxic conditions, and ^&^ *p* < 0.05 and ^&&&^ *p* < 0.001 compared to 150 IEQ/cm^2^ under hypoxic conditions.

In order to evaluate the effects of cell density on the inflammatory response induced during hypoxia, we measure the relative mRNA expression of two inflammation markers, Cox-2 and Ho-1, as well as the secretion of IL-6. We observed that hypoxia induced a significant upregulation of both Cox-2 (Figure 4(a), *p* < 0.05) and Ho-1 (Figure 4(b), *p* < 0.05) mRNA expression compared to normoxia, and these increases in gene expression appear to occur independently of islet density. Interestingly, this response appears to be significantly greater in the control cells compared to the higher densities. Furthermore, IL-6 secretion did not appear to be influenced by the change in PO_2_ to hypoxic levels at any cell density. However, IL-6 secretion is seemingly controlled independently of PO_2_, as a significant increase was observed for both of the higher densities under normoxic conditions compared to the control cells as well as under hypoxic condition for the 300 IEQ/cm^2^ density compared to the control (*p* < 0.05) (Figure 4(c)).

## 4. Discussion

In the present study, we have shown that the combination of hypoxia and increased islet density/confinement, which are the typical conditions in a bioartificial pancreas, causes an increased level of islet cell death. Indeed, when the recommended culture density was doubled (300 IEQ/cm^2^) or multiplied by four (600 IEQ/cm^2^) and maintained under hypoxic conditions, the overall survival of the islets was very poor, with an observed activation of apoptosis and necrosis factors as well as loss of function and proinflammatory cytokine secretion. To our knowledge, this is the first published study demonstrating the specific effects of hypoxia on confined islet cell survival and function.

In order to effectively and efficiently combat diabetes, it is essential that bioartificial pancreases maintain a sufficient number of viable, fully functioning islets cells. Based on clinical hepatic islet transplantations, 10,000 IEQ/kg is needed to reach normoglycaemia [1]. Unfortunately, it has been estimated that approximately 40% of the transplanted islets will die during the instant blood mediated inflammatory reaction (IBMIR) [23] and another 10 to 20% will die during vascularization [24]. These losses can be avoided when using a bioartificial pancreas. However, in contrast with liver transplantation, islets will never be vascularized in a device due to the immune-isolation required. Therefore, no oxygen or nutrients will be delivered directly to islets by the vessels, because only the device can be vascularized [3, 25]. Oxygen will be delivered from the surrounding vessels to the device or via diffusion in the case of nonvascularized devices and will then need to diffuse into the cells, resulting in low cellular PO_2_ (around 15 mmHg in the case of nonvascular devices) [15].

Notably, in an alternative study, a *β*-air II device, with PO_2_ between 304 and 198 mmHg in the gas chamber and more than 2,000 IEQ/cm^2^, was used once a day to increase oxygenation of encapsulated islets in rats [26]. However, a previous report suggests that such a fluctuation in oxygen, going from hyperoxia to hypoxia, may cause oxygen toxicity [27]. Moreover, the device is not completely independent from external intervention, and it is possible that the oxygen injected into the chamber can actually generate ROS [28]. It is likely that the complications caused by such oxygenating devices will prevent their widespread clinical use.

Thus, in order to compensate for islet cell death in bioartificial pancreases, a higher number of cells are typically cultured in the device. Our results in the present study show that when the number of islets was doubled or increased four times the recommended density for islet culture, the oxygen availability decreased. In fact, the oxygen concentration dropped from 15 mmHg to 7 mmHg at the highest cell density, a change that will undoubtedly have repercussions in terms of islet survival. Interestingly, it has been shown that the cells consuming oxygen will actually create an oxygen gradient [21] further depriving other cells from oxygen. Moreover, under hypoxic conditions, we observed the effects of cell density to be amplified, leading to cell death and loss of function in less than 24 h. Zheng et al. [29] previously demonstrated that adaptation to hypoxia is difficult for normal, untreated islets, suggesting that it could be even more difficult for transplanted/cultured islets. To this end, we have shown that hypoxic conditions combined with confinement triggered HIF-1*α* transcription, which was also correlated with activation of Cox-2 (involved in inflammatory process) and Ho-1 (involved in oxidative stress) mRNA expression [19]. These data imply that the cells are at least attempting to activate their endogenous defense system against the lack of oxygen. Notably, the increased expression of both Cox-2 and Ho-1 in the higher density cells was significantly lower than the increase observed in the cells cultured at a normal density and appears to be insufficient, leading to an increased rate of apoptosis in the more confined cells. We also observed that secretion of IL-6 is increased as the level of cell confinement increased, regardless of oxygen availability. The role of IL-6 during islet cell survival is controversial in the literature. IL-6 is secreted as a response to cell stress and some studies have suggested that it is deleterious for islet cells [19], while others indicate that it is beneficial for their function [30]. Here, IL-6 secretion appears to be related to confinement only. However, it is possible that the 24-hour time point used in this study may have been too early to see the effects of hypoxia on IL-6 secretion. The increase in IL-6 secretion in the 300 and 600 IEQ/cm^2^ could be also attributed to the paracrine and autocrine effect IL-6 has on its own secretion. Thus, the more cells there are in a limited space, the more IL-6 will be secreted as each islet will be more able to induce the surrounding cells. As this cytokine is known to play a significant role in inflammation [31], its secretion in the more confined cells could affect the recruitment and activation of host macrophages and leukocytes around the device, potentially increasing fibrosis and preventing good vascularization.

In terms of islet cell function, the decrease we observed in cellular ATP was enhanced under hypoxia, a phenomenon that is likely due to ATP leakage, which has been described in cases of organ ischemia [32–34]. Under low PO_2_, the tricarboxylic acid cycle cannot be used and the switch for anaerobic respiration is triggered, but this alternative pathway produces a much lower amount of ATP. It was, therefore, not surprising that the level of ATP was significantly decreased during hypoxia. This loss of ATP was also correlated with a decrease in the cell's ability to respond to glucose stimulation. Notably, ATP and insulin are strongly correlated, and the conversion of proinsulin to insulin and C-peptide is, at least in part, stimulated by ATP-dependent processes [35]. This relationship likely plays a significant role in the decrease function observed in the cells cultured at a higher density under hypoxic conditions.

## 5. Conclusion

We have shown that islets in a confined environment and under hypoxia suffer from hypoxia-induced apoptosis, oxidative stress, and inflammation, and are nonfunctional after only 24 hours of incubation. In order to improve islet survival in a bioartificial pancreas, the effects of hypoxia need to be reduced, as well as the inflammatory response, in order to improve islet survival and function after transplantation. While this study highlights the need for these changes, additional work is necessary in order to develop new strategies to improve the outcome of islet transplantation in patients with diabetes.

## Figures and Tables

**Table 1:** qPCR primers.

Primers	Reference
Rn_Rnr1_1_SG QuantiTect primer assay	QT00199374
Rn_Ppia_1_SG QuantiTect primer assay	QT00177394
Rn_Rplp1_2_SG QuantiTect primer	QT01745625
Rn_Hif1a_1_SG QuantiTect primer assay	QT00182532
Rn_Ptgs2_1_SG QuantiTect primer assay	QT00192934
Rn_Hmox1_1_SG QuantiTect Primer assay	QT00175994

**Table 2:** Oxygen measurements at time 0 (*t* = 0) and after 24 hours (*t* = 24 h) for each islet culture density (150, 300, and 600 IEQ/cm²) under normoxic (PO_2_ 160 mmHg) and hypoxic (PO_2_ 15 mmHg) conditions. The difference between *T*_0_ and *T*_24_ is represented by the *p* values. The difference between densities within the same condition is represented by *∗*.

	PO_2_ 160 mmHg			PO_2_ 15 mmHg		
	150 IEQ/cm²	300 IEQ/cm²	600 IEQ/cm²	150 IEQ/cm²	300 IEQ/cm²	600 IEQ/cm²
*t* = 0	143.8 ± 3.8	143.1 ± 4.7	133.9 ± 5.1	150.1 ± 3.2	142.4 ± 3.3^*∗*^	133.0 ± 4.6^*∗*^
*t* = 24 h	121.6 ± 9.7	99.5 ± 12.7^*∗*^	80.5 ± 8.7^*∗∗∗*^	14.3 ± 6.2	10.5 ± 5.1	7.6 ± 3.2
*p* value	0.03	0.00098	0.00007	0.034	0.000010	0.00000

^*∗*^ *p* < 0.05 and ^*∗∗∗*^ *p* > 0.001.

## Nomenclature

PO_2_: Oxygen partial pressure
ATP: Adenosine triphosphate
Rplp1: Ribosomal protein P1
Ppia: Peptidylprolyl isomerase A
Rnr1: RNA ribosomal 1
Hif-1*α*: Hypoxia-inducible factor 1 alpha
Ptgs2/Cox-2: Prostaglandin-endoperoxide synthase 2
Hmox1/Ho-1: Heme oxygenase 1
ROS: Reactive oxygen species
IEQ: Islet Equivalents.
